# Case Report of Meconium Peritonitis: A Rare Cause of Non-immune Hydrops Fetalis

**DOI:** 10.7759/cureus.72860

**Published:** 2024-11-01

**Authors:** Radhika Maddali, Carmen Alvarez-Gell, Palanikumar Balasundaram, Mohamed Sakr

**Affiliations:** 1 Department of Neonatal-Perinatal Medicine, Montefiore Medical Center, Bronx, USA; 2 Department of Pediatrics, Flushing Hospital Medical Center, Flushing, USA; 3 Division of Pediatrics, The Children’s Hospital at Montefiore and Albert Einstein College of Medicine, Bronx, USA; 4 Department of Pediatrics, University of Illinois College Medicine Rockford, Rockford, USA; 5 Department of Pediatrics, Division of Neonatology, Mercy Health, Javon Bea Hospital-Riverside, Rockford, USA; 6 Department of Neonatology, The Children's Hospital at Montefiore, Bronx, USA

**Keywords:** fetal ascites, meconium peritonitis, neonatal pneumoperitoneum, non-immune hydrops fetalis, torch infections

## Abstract

Meconium peritonitis (MP) as a cause of non-immune hydrops in neonates is rarely reported. We present a case of a 35-week gestational-age male neonate diagnosed with hydrops secondary to MP. Antenatal scan at 34 weeks and five days revealed features of fetal hydrops, and the prenatal workup did not reveal the etiology. Postnatal imaging confirmed MP with abdominal calcifications, and the neonate developed pneumoperitoneum due to intestinal perforation. Emergency laparotomy revealed diffuse MP without a definitive site of perforation. An ileostomy was created and closed before discharge. Follow-up showed normal growth and development, emphasizing the importance of early diagnosis and multidisciplinary management in such rare cases. MP, though rare, should be considered in the differential diagnosis of non-immune hydrops fetalis (NIHF), particularly in the presence of fetal ascites.

## Introduction

Meconium peritonitis (MP) is a rare condition resulting from intrauterine bowel perforation, leading to leakage of meconium into the peritoneal cavity and subsequent chemical peritonitis [1]. Early prenatal diagnosis and ongoing fetal well-being monitoring, along with the exclusion of chromosomal abnormalities; congenital toxoplasmosis, rubella cytomegalovirus, herpes simplex, and HIV (TORCH) infections; and cystic fibrosis, are crucial for improving prognosis [2,3]. MP is a rare cause of non-immune hydrops fetalis (NIHF), with only a few cases reported in the literature [4]. We present a rare case of a neonate diagnosed antenatally with NIHF who was later found to have MP secondary to intestinal perforation.

## Case presentation

A 3.19 kg male infant was born via cesarean section at 35 weeks gestational age to a 28-year-old third gravida woman with no history of consanguinity. During a routine prenatal visit, the mother was noted to have a fundal height larger than expected for gestational age. Antenatal ultrasonography at 34 5/7 weeks gestational age revealed a single live intrauterine fetus with an amniotic fluid index (AFI) of 22.4 cm (85th percentile), suggestive of borderline high AFI. Additionally, features of fetal hydrops were observed, including fetal ascites, mild pericardial effusion, left pleural effusion, visible skin edema in the abdomen and thorax, and subcutaneous scalp edema. The estimated fetal weight was 3.39 kg (>97th percentile), and the abdominal circumference was > 97th percentile. Aneuploidy screen at 17 weeks was negative, and the anatomy scan at 21 weeks of gestation was normal. The mother’s hemoglobin was 10.5 g/dl, and her blood pressure remained normal. Antenatal workup for the hydrops, including middle cerebral artery peak systolic velocity; rubella, toxoplasmosis, cytomegalovirus, parvovirus, and syphilis serologies; and fetal echocardiography, were normal. Her carrier screen for cystic fibrosis was negative. Due to nonreassuring fetal heart tracing, a cesarean section was performed at 35 weeks gestational age. The infant had appearance, pulse, grimace, activity, and respiration (APGAR) scores of 9/9, did not require resuscitation in the delivery room, and was transferred to the neonatal intensive care unit (NICU) for further evaluation. At admission, the infant had a normal physical examination with a birth weight of 3190 g (95th percentile), length of 45.5 cm (42nd percentile), and head circumference of 33 cm (36th percentile). However, at 30 minutes of life, he had increased work of breathing, and continuous positive airway pressure (CPAP) was initiated. 

**Figure 1:**
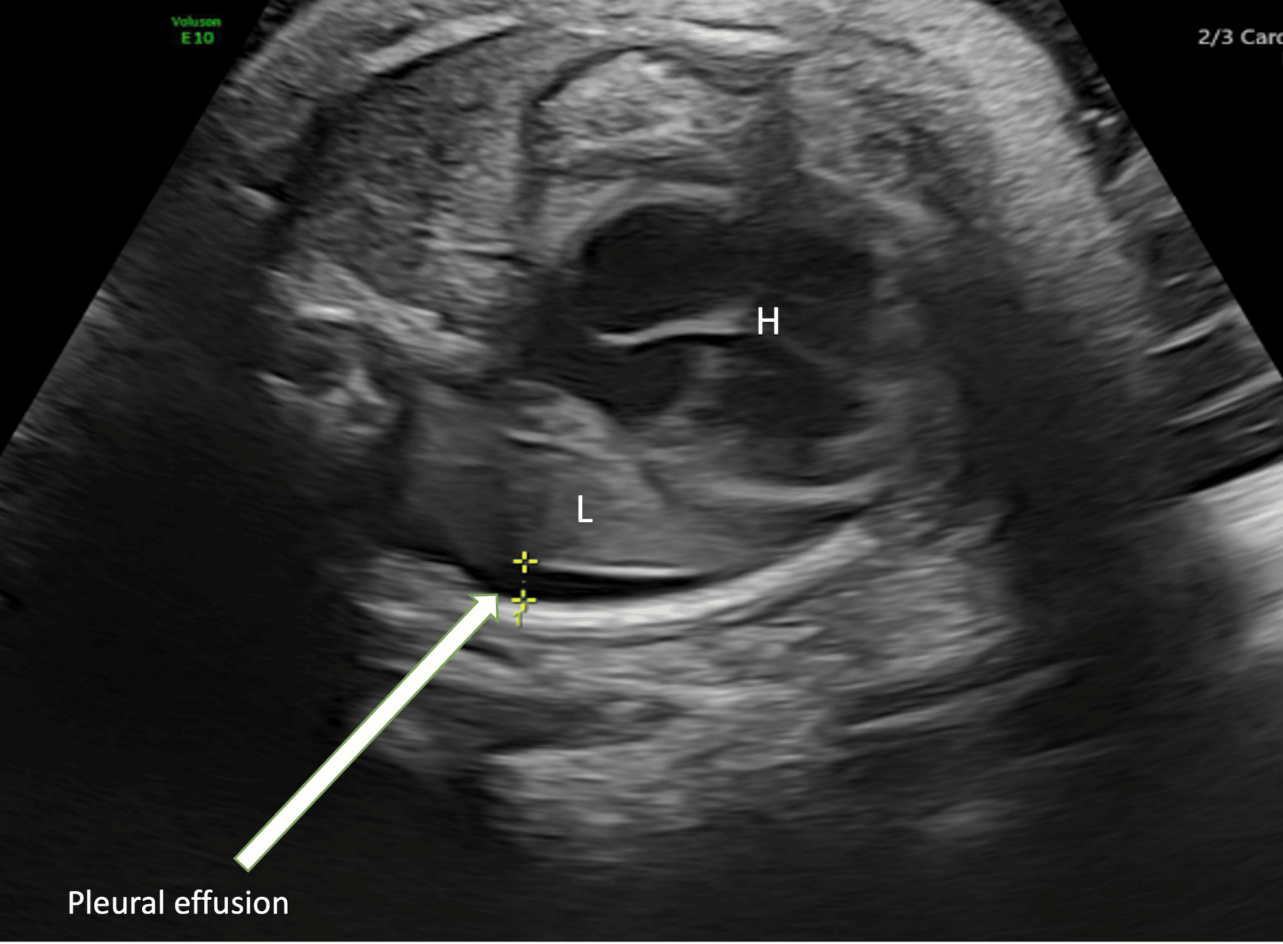
Chest ultrasound showing pleural effusion L: Lung; H: heart

Investigations showed the infant’s blood group to be O with Rh positive and direct antiglobulin test (DAT) negative, and no features suggestive of hemolysis in the peripheral smear. The mother’s blood group was B with Rh positive and DAT negative, ruling out the possibility of Rh and ABO incompatibility. The admission hemoglobin was 12.9 g/dl, and renal and liver function tests were within the normal limits. A chest X-ray suggested increased vascular markings suggestive of transient tachypnea of newborn and minimal blunting of costophrenic angles. Ultrasound of the chest showed small bilateral pleural effusions (Figure 1). 

**Figure 2:**
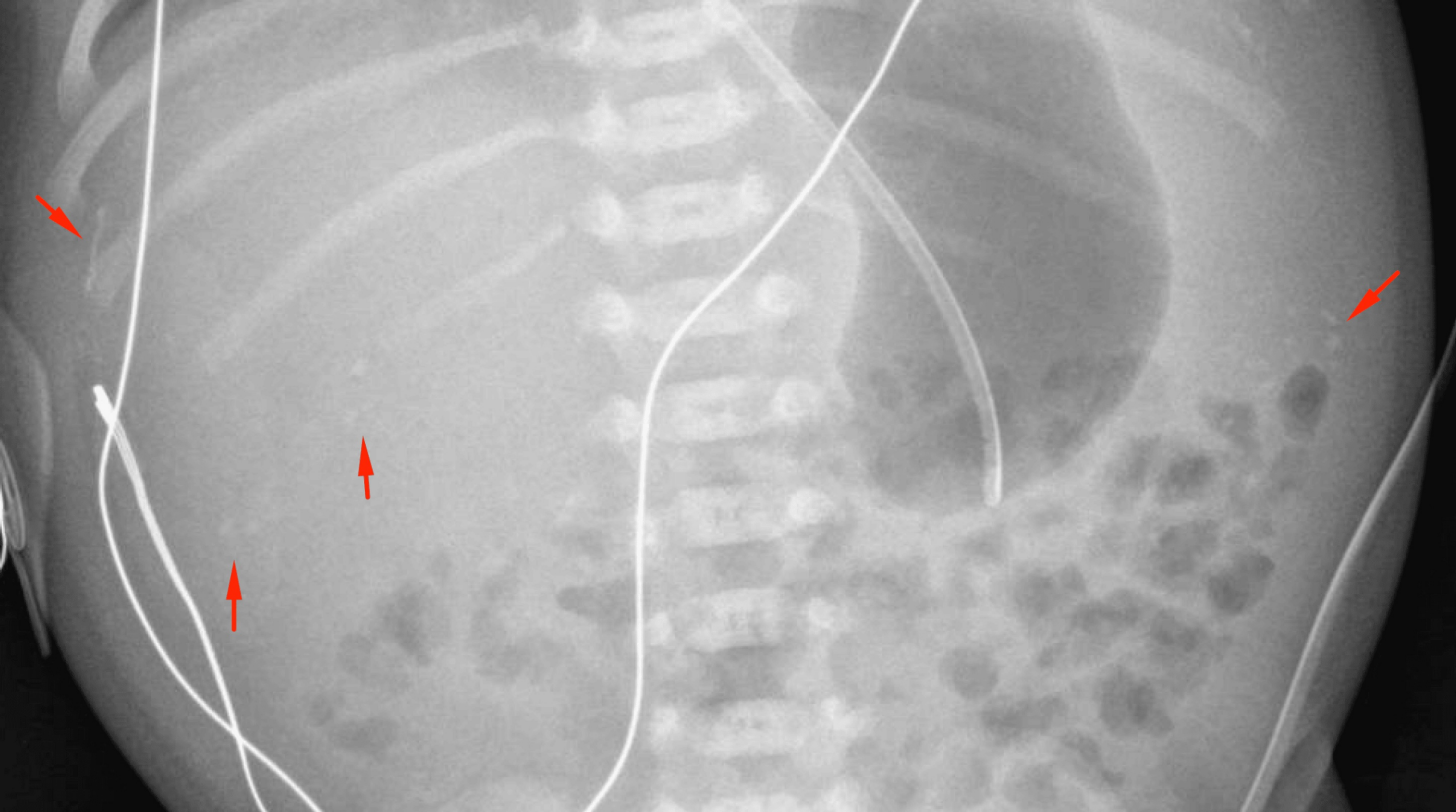
Abdominal X-ray showing multiple calcifications over the abdominal cavity consistent with meconium peritonitis

Abdominal X-ray (AXR) (Figure 2) showed multiple calcified foci over the abdomen suspicious of MP and no bowel obstruction. Abdominal ultrasound showed calcifications consistent with MP with no evidence of ascites.

**Figure 3:**
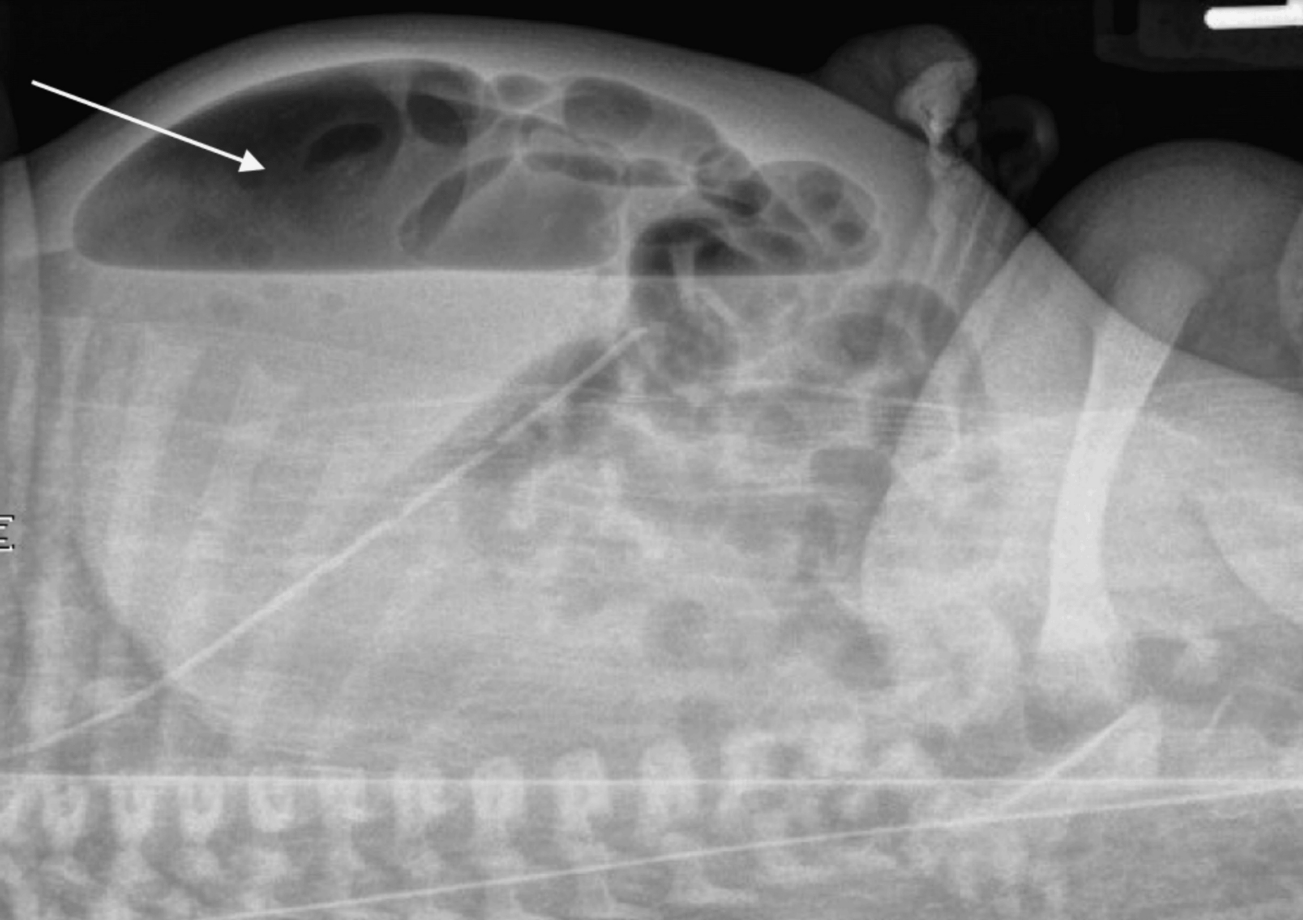
Abdominal X-ray (cross-table lateral view) showing amorphous air and fluid collection suggestive of hydro-pneumoperitoneum with diffuse calcifications indicating ceconium peritonitis

The infant was kept nil-per-os, and intravenous fluids were initiated. On the second day of life, the clinical exam showed increasing abdominal distension prompting a repeat AXR, which showed multiple calcifications throughout the abdominal cavity and worsening focal distention of bowel loops in the right abdomen with air-fluid levels. Sump decompression was initiated. A follow-up AXR showed hydro-pneumoperitoneum (Figure 3).

An exploratory laparotomy was performed and immediately revealed MP with a large amount of green-tinged fluid upon entry into the peritoneal cavity, followed by an additional gush of meconium and green-tinged fluid upon manipulation of the bowel. Diffuse abdominal calcifications were noted, but no definitive perforation, malrotation, loculated meconium, or pseudocyst were identified. A proximal loop ileostomy was created. Oral feeds were started on a postoperative day (POD) 5, and the infant reached full feeds by POD 7. The infant had bacteremia during the NICU stay and was treated with antibiotics for 14 days, and closure of ileostomy was performed on the 81st postnatal day. The infant was discharged on the 93rd postnatal day. On follow-up at four months of age, he was on exclusive breastfeeding with normal growth and development. 

## Discussion

NIHF is defined as the presence of two abnormal fetal fluid collections in the absence of red cell alloimmunization. NIHF currently accounts for 90% of cases of hydrops [5]. A rare etiology of NIHF is MP, sterile chemical peritonitis caused by antenatal perforation of the intestinal tract and subsequent leakage of meconium into the peritoneal cavity [2].

The etiology of MP may vary, but essentially it is due to intestinal bowel perforation secondary to a mechanical obstruction like volvulus, intestinal atresia, intussusception, internal hernia, congenital bands, or meconium ileus with or without cystic fibrosis [6]. Peritoneal macrophages play a key role in the pathophysiology of MP, leading to dense inflammation in response to meconium and secretion of cytokines like tumor necrosis factor-alpha. Inflammation, along with exudates, can completely seal off the perforation or form a large cyst if the perforation is not sealed [7]. MP is classified into three main types: fibro-adhesive, cystic, and generalized. In the fibro-adhesive form, a fibrous mesh or membrane forms around the intestines secondary to an inflammatory process. The cystic type results from localized accumulation of ascitic fluid with intestinal and omental adhesions that lead to pseudocyst formation. In the generalized type, diffuse calcium plaques form in the peritoneal cavity [8]. Intestinal enzymes precipitate calcium salts with fatty meconium molecules, resulting in calcifications, the pathognomonic sign of MP [9]. Our case exemplifies a generalized MP with scattered calcifications in the abdominal radiograph. 

**Figure 4:**
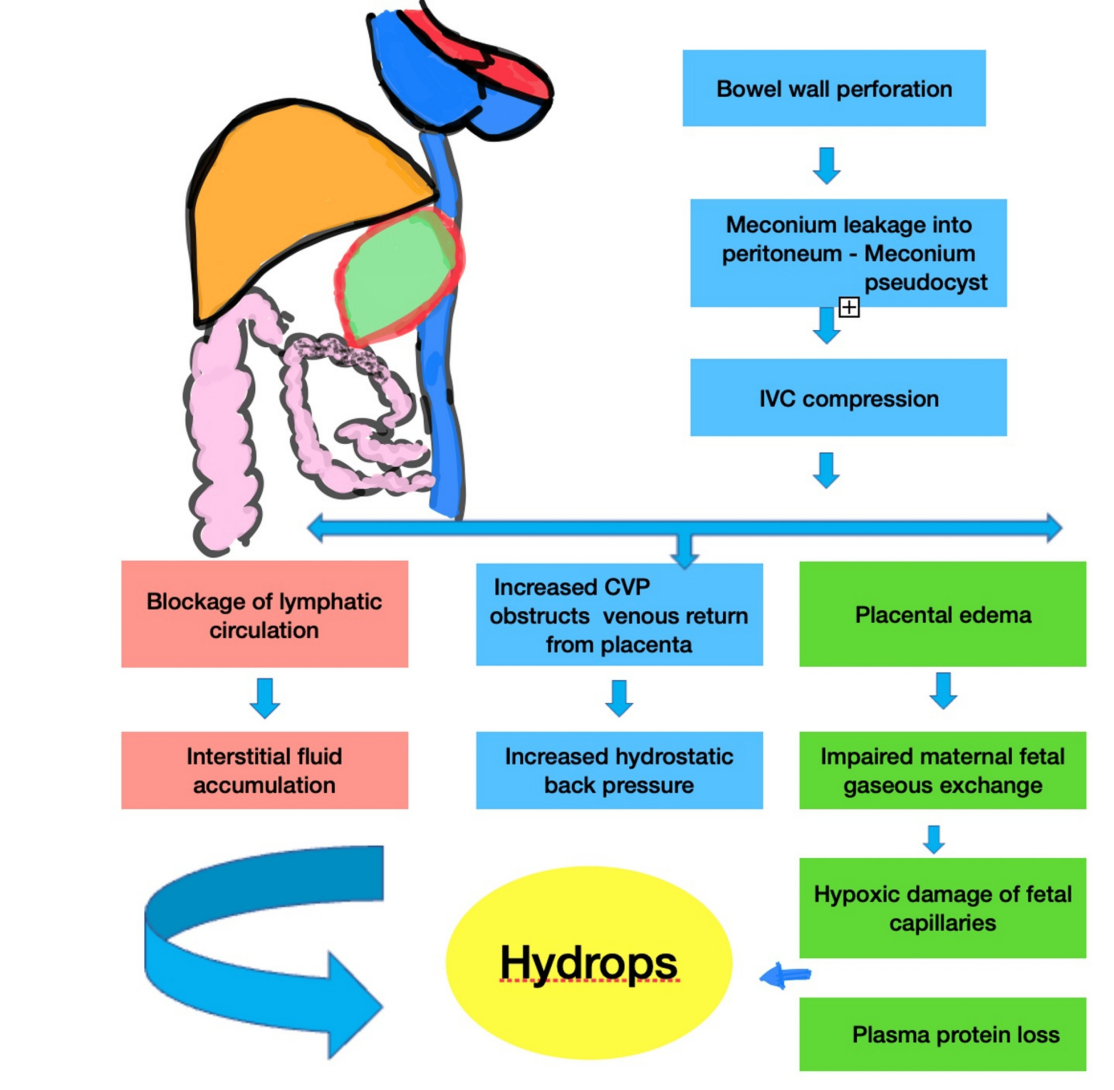
Pathophysiology of meconium peritonitis and subsequent hydrops IVC: Inferior vena cava; CVP: central venous pressure

**Figure 5:**
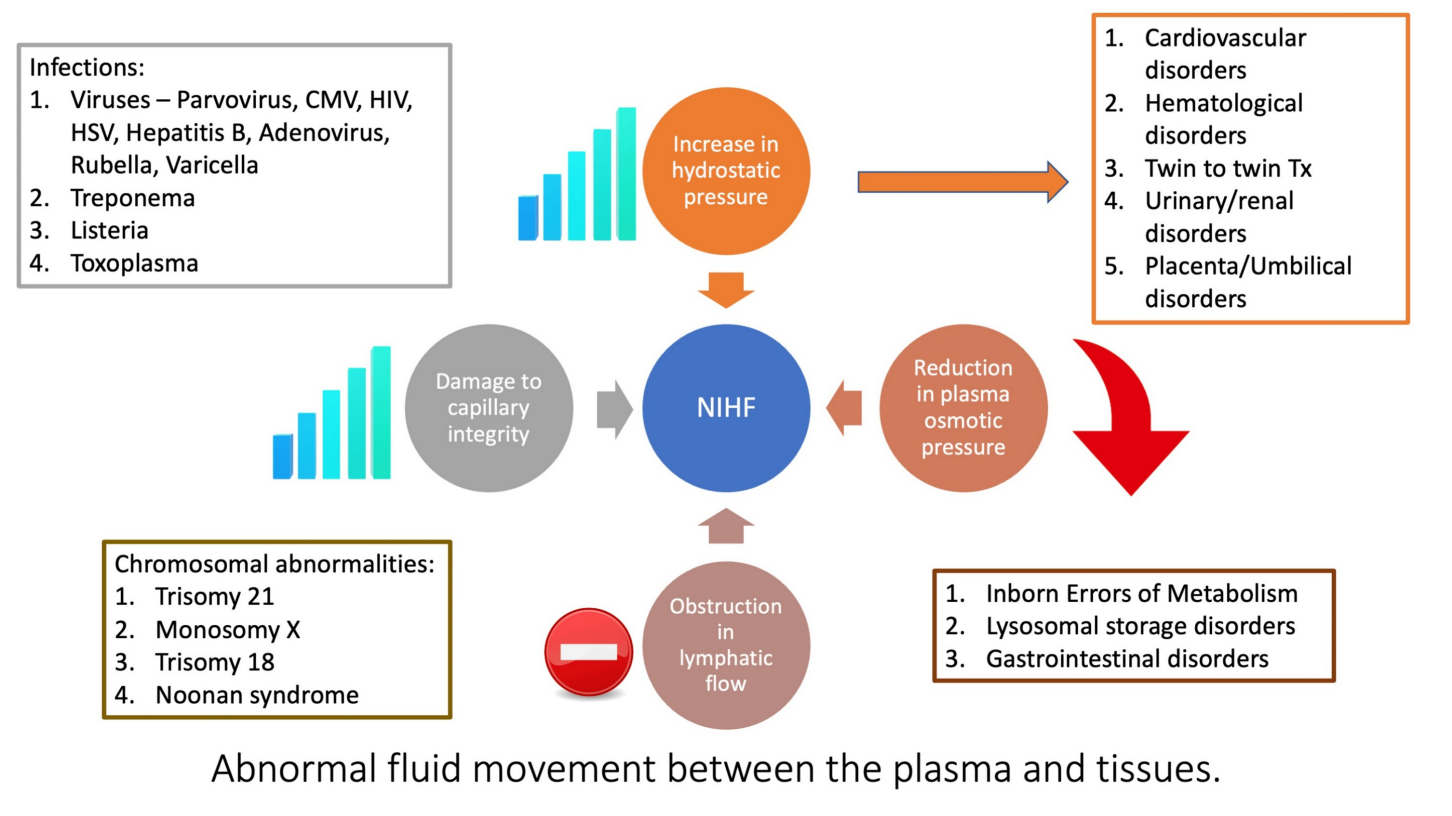
Pathophysiology of non-immune hydrops fetalis (NIHF) NIHF: Non-immune hydrops fetalis; CMV: cytomegalovirus; HSV: herpes simplex virus; HIV: human immunodeficiency virus; Tx: transfusion

The basic mechanism for the formation of hydrops is due to an accumulation of interstitial fluid, increased hydrostatic back pressure, or plasma protein loss. In many circumstances, this imbalance can be due to infections, chromosomal abnormalities, placental disorders, twin-to-twin transfusions, or disorders in different organ systems, including the heart, blood, or kidneys (Figures 4-5) [10]. The placental pathology in this case reported a large placenta, >90-95th percentile, which can impair the maternal-fetal gas exchange and cause plasma protein loss.

Prenatal diagnosis of MP with ultrasound can show fetal ascites (the most common finding), dilated bowel loops, calcification, echogenic bowel, and polyhydramnios. Postnatal abdominal radiography may confirm these findings and determine the extent of dilation or obstruction. On physical exam, neonates may present with abdominal distension, bilious emesis, delayed meconium passage, or respiratory distress [11]. In this case, we witnessed an acute development of increasing abdominal distension. 

Neonates with worsening hydrops who are >34 weeks are likely to require delivery. Unless there are maternal indications, cesarean section has not been found to decrease morbidity when compared to vaginal delivery [12]. Postnatal care in the delivery room requires a skilled neonatal resuscitation team. Additional interventions like intubation, chest tube placement, paracentesis, and pericardiocentesis may be needed on a case-to-case basis. Postnatal evaluation with laboratory work can include a complete blood picture, type, and screen and DAT test, a comprehensive metabolic panel including liver function tests, blood culture, and newborn screen. If there is cardiac involvement, electrocardiogram and echocardiogram are warranted, along with a cardiology consult for further evaluation. If pleural fluid is collected, cell count, total protein, lactate dehydrogenase (LDH), triglyceride level, and culture with the Light’s criteria can provide useful information [13]. Infectious workup should include parvovirus IgM/IgG, toxoplasma IgM/IgG, rubella, urine cytomegalovirus, herpes simplex polymerase chain reaction (PCR), HIV PCR, and syphilis testing [2]. Genetic workup should be done if not pursued prenatally. Ultrasound and radiographs of the chest and abdomen should be included in the workup. Treatment of MP can be conservative or surgical based on the clinical findings and stratification of imaging findings [9,14]. Close monitoring with serial abdominal exams and ancillary imaging can define the need for surgical care.

Our case presents a 35-week neonate with ascites, pleural effusion, skin edema, and borderline high AFI visualized in the last prenatal ultrasound before delivery. This patient developed significant abdominal distension and air-fluid levels on AXR with pneumoperitoneum, requiring emergency laparotomy on the second day of life. Therefore, it is essential to involve the pediatric surgery team early on and discuss clinical changes in the patient’s course as a multidisciplinary team. 

## Conclusions

MP leading to NIHF is a rare presentation in neonates. An early prenatal diagnosis of MP leads to anticipated management and treatment, improving the patient's overall outcome. Thus, antenatally, if the fetus has ascites as the predominant manifestation, and progresses to hydrops, other findings suggestive of MP, such as dilated bowel loops or calcifications, should be looked for, and prompt postnatal management results in a good outcome. Postnatal testing can help illustrate underlying factors that may contribute to the overall picture, and thus, management may vary on a case-to-case basis. The treatment of MP requires a multidisciplinary team and rapid execution of a contingency plan if surgical intervention is indicated.
